# Complementary Activities of Host Defence Peptides and Antibiotics in Combating Antimicrobial Resistant Bacteria

**DOI:** 10.3390/antibiotics12101518

**Published:** 2023-10-06

**Authors:** Patrick R. Lennard, Pieter S. Hiemstra, Peter H. Nibbering

**Affiliations:** 1 Centre for Inflammation Research, Institute for Regeneration and Repair, University of Edinburgh, Edinburgh EH16 4UU, UK; 2 Institute of Immunology and Infection, School of Biological Sciences, University of Edinburgh, Edinburgh EH9 3FE, UK; 3 PulmoScience Lab, Department of Pulmonology, Leiden University Medical Centre, Leiden 2333, The Netherlands; 4 Department of Infectious Diseases, Leiden University Medical Centre, Leiden 2333, The Netherlands; nibbering@hhvbiotech.com

**Keywords:** host defence peptide, antimicrobial peptide, antibiotics, synergism, antimicrobial resistance

## Abstract

Due to their ability to eliminate antimicrobial resistant (AMR) bacteria and to modulate the immune response, host defence peptides (HDPs) hold great promise for the clinical treatment of bacterial infections. Whereas monotherapy with HDPs is not likely to become an effective first-line treatment, combinations of such peptides with antibiotics can potentially provide a path to future therapies for AMR infections. Therefore, we critically reviewed the recent literature regarding the antibacterial activity of combinations of HDPs and antibiotics against AMR bacteria and the approaches taken in these studies. Of the 86 studies compiled, 56 featured a formal assessment of synergy between agents. Of the combinations assessed, synergistic and additive interactions between HDPs and antibiotics amounted to 84.9% of the records, while indifferent and antagonistic interactions accounted for 15.1%. Penicillin, aminoglycoside, fluoro/quinolone, and glycopeptide antibiotic classes were the most frequently documented as interacting with HDPs, and *Staphylococcus aureus*, *Pseudomonas aeruginosa*, *Escherichia coli*, and *Enterococcus faecium* were the most reported bacterial species. Few studies formally evaluated the effects of combinations of HDPs and antibiotics on bacteria, and even fewer assessed such combinations against bacteria within biofilms, in animal models, or in advanced tissue infection models. Despite the biases of the current literature, the studies suggest that effective combinations of HDPs and antibiotics hold promise for the future treatment of infections caused by AMR bacteria.

## 1. Introduction

**Figure 1:**
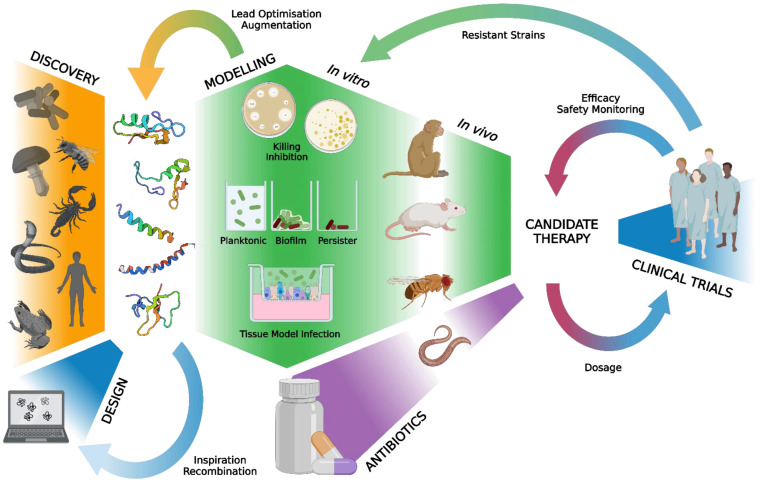
Schematic representation of the development of HDPs and their combination with antibiotics as novel approaches to combat AMR bacterial infections. HDPs are identified from various species or rationally designed de novo for optimal antimicrobial activity. Initial modeling of their activity against bacteria is conducted in vitro, assessing minimum antibacterial concentrations, cytotoxicity, activity against various bacterial stages (i.e., biofilm-established and persister cultures), and activity in host tissue infection models. Synergistic actions between HDPs and established antibiotics can also be conducted before progression to in vivo pre-clinical models. Combined synergistic, effective, and safe therapies may then progress to clinical trial stages, where safety and efficacy feedback is used to optimise therapies (red-blue arrows). Created with BioRender.com.

Antimicrobial resistance is a multifactorial and impending global health crisis responsible for an estimated 5 million deaths annually and is likely to surpass 10 million deaths annually by 2050 [1,2]. Efforts to combat the progressively increasing burden of antimicrobial resistance are limited by the decreasing efficacy of current antimicrobial treatment, the development of multi-drug resistant pathogens, and the limited development of novel antimicrobial agents. As such, many approaches to combat the imminent threat of infection by antimicrobial resistant (AMR) micro-organisms have been proposed, including the development of vaccines and alternative therapies against resistant and tolerant bacteria. The latter includes the development of new agents to bolster our antibiotic arsenal, such as those that improve and synergise with conventional antibiotics [3]. The use of antibiotics alone to combat AMR bacterial infections is insufficient in the wake of the wide onset development of resistance and the difficulty in treating chronic and deep-seated infections. AMR bacteria can colonise the host, form biofilms and persisters, reside within host cells, and establish chronic infections that evade treatment. The current pipeline for the development of new antibiotics is a lengthy and complex process. It includes identifying new agents and targets, investigating their mechanisms of action, assessing across a range of in vitro and in vivo models, and conducting clinical trials (illustrated in Figure 1 for the development of HDPs and combinations with antibiotics). While repurposing existing drugs offers a means to partially circumvent this process [4,5], the antibiotic toolkit is running dry, and resistance to last-resort antibiotics, such as carbapenems and colistin, is developing rapidly [6].

Host defence peptides (HDPs, also known as antimicrobial peptides (AMPs)) have been proposed as alternatives and supplements to established antibiotics [7]. HDPs are mostly cationic peptides expressed by host cells and display broad-spectrum antimicrobial activity against bacteria, fungi, and viruses. They hold promise as infection treatment candidates as they combine antibacterial (bactericidal, anti-biofilm, and toxin-neutralizing) capabilities with the additional ability to modulate the host immune defence. HDPs are conventionally less than 50 amino acids in size and have a charge between +2 and +9 at physiological pH [7]. A key differentiator between HDPs and antibiotics is their alternative mode of bactericidal activity, i.e., the ability of HDPs to destabilise and subsequently destroy the bacterial plasma membrane, including that of AMR bacteria [8]. However, some HDPs penetrate bacteria and mediate killing via intracellular targets such as bacterial DNA and ribosomes [9,10]. A wealth of HDPs have been identified across the diverse kingdoms of life, produced by bacteria, fungi, plants, and animals as an integral first-line defence against pathogens and competing species [11]. Under the growing threat of AMR, HDPs are highlighted as strong contenders for preclinical investigation, and various HDPs are being considered or have already been evaluated in clinical studies for the treatment of a wide variety of infectious and inflammatory diseases [12]. However, HDPs have limitations including—but not limited to—their peptidic nature and cytotoxicity. Both cathelicidins and defensins—the two most investigated families of HDPs—can be toxic to different cells and tissue, induce adverse stimulation of, e.g., mast cells [13,14], have reduced activity in the presence of serum [15,16], and are susceptible to degradation by host and bacterial proteases [17,18,19]. While HDPs hold promise due to their dual activity against AMR bacteria, further improvement is required to mitigate their potential adverse effects and advance past pre-clinical stages, including the advanced formulation and augmentation of peptides [20] and their combination and conjugation with established and novel nonpeptide antibiotics [21,22].

The combination of novel HDPs and antibiotic therapies, targeted against broad and distinct bacteria traits, may be the key to overcoming the inefficacy and failure of conventional antibiotic therapy against AMR bacteria. Conventional antibiotics principally target bacteria by disrupting the bacterial cell wall and its synthesis and targeting intracellular components. General classes of antibiotics target the following components: aminoglycosides (AG), amphenicols (AM), macrolides (ML), and tetracyclines (TC) bind and inhibit bacterial ribosomes; carbapenems (CP), cephalosporins (CS), glycolipids (GP), penicillins (PC), and phosphoglycolipids (PG) inhibit peptidoglycan synthesis; fluoro/quinolones (FQ) target DNA gyrase; and rifamycins (RF) target bacterial RNA polymerase [23]. Both polymixins (PM) and polypeptide (PP) antibiotic classes typically inhibit bacteria by disrupting membrane integrity [24], similar to HDPs. Most HDPs can disrupt and destabilise the bacterial membrane, both directly killing bacteria and removing a barrier for other agents, e.g., antibiotics, to act upon intracellular components of susceptible bacteria. Therapy with HDPs could be advantageous by targeting alternate bacterial mechanisms and due to several of their key activities: reduction of functional concentrations, optimization of safety in vivo, extension of antibacterial activity spectra, re-sensitization to antibiotics, and eradication of biofilms and persisters. The combination of HDPs with antibiotics has been investigated extensively, from defining synergy and optimal peptide characteristics to systematically modeling HDP–antibiotic interactions using deep learning [5,25]. Individual peptides and antibiotics are often the foci of in vitro investigation through sequential augmentation and development due to the difficulty, labour, and cost of assessing arrays from the known peptide and antibiotic libraries. Similarly, the activities of these agents are most often assessed against the ESKAPE pathogens (i.e., *Enterococcus faecium*, *Staphylococcus aureus*, *Klebsiella pneumoniae*, *Acinetobacter baumannii*, *Pseudomonas aeruginosa*, and *Enterobacter* species) due to their frequent resistance development. Such a concentration of efforts, in addition to their assessment in acellular conditions, limits our understanding of the diverse HDP–antibiotic synergies that may exist and the interactions agents may have with the host tissue and immune system against AMR bacteria [26].

While several studies have recently reviewed the literature on synergistic activity between HDPs and antibiotics [21,27], scrutiny is rarely turned toward discrepancies in the literature. Studies vary greatly in their assessments of synergy, experimental methods used, and the pathogens against which antibiotic combinations are assessed. Further, AMR bacteria and bacteria within biofilms are also understudied within the literature despite their clinical importance as targets of synergistic therapy. As clinical infections involve planktonic bacteria and their counterparts in biofilms, understanding the effects of HDP–antibiotic combinations on these stages in the bacterial life cycle is integral to the clinical progression of combination therapies against AMR bacteria. Here, we present a compilation and in-depth analysis of the combinatory antibacterial activities of HDPs with antibiotics reported against AMR bacteria. In addition, we analysed the methods used for assessing HDP–antibiotic synergy, the antibiotic classes assessed, the pathogens targeted, and the experimental models utilised across the literature. This review highlights the discrepancies across the literature and provides a resource to address such gaps and expedite the development of synergistic HDP–antibiotic therapies against AMR bacteria.

## 2. Methods

### 2.1. Literature Strategy

Published articles on using HDPs in combination with established antibiotics against AMR bacteria were identified through computerised literature searches using Google Scholar, Microsoft Academic, PubMed, Web of Science, and SCOPUS databases. Searching the databases, the following search terms were used: “antibiotic” AND (“antimicrobial peptide” OR “host defence peptide”) AND (“synergy” OR “complementary” OR “tandem”) AND (“resistant bacteria”) AND (“combination” OR “modified” OR “augmented” OR “conjugate”)]. The following search term was excluded from searches due to superfluous results: “phage”. Other relevant papers were identified with citation trackers and integrated throughout the review period until submission (i.e., from August 2021 to August 2023).

### 2.2. Eligibility Criteria

Only original, peer-reviewed research articles, available in full-text format in English, were compiled from the database search results. Studies were limited to those explicitly reporting antibacterial interactions between HDPs (or their derivatives) and antibiotics against resistant bacteria. Records were excluded if they did not report: (i) HDPs, or peptides rationally designed from HDPs, and conventional antibiotics (i.e., clinically approved medications); and (ii) the combined use of HDPs or their derivatives with antibiotics against AMR bacterial strains.

### 2.3. Study Selection and Data Extraction

Studies were extracted from search databases, and eligibility was assessed for each article. Data from each study were verified for consistency and accuracy and then entered into a database for variables to be stratified. The risk of bias in the included studies was not formally assessed. Abstracted information included: first author, last author, year of publication, origin peptide, species of origin, applied peptide, antibiotics assessed, class(es) of antibiotics, method of application, bacteria assessed, assessment against bacterial biofilms, assessment of synergism, and experimental model used. Studies that used an accurate assessment of synergistic activity between agents were selected based on their use of a fractional inhibitory concentration index (FICI) or a similar derivative of the formula below:

$$FICI\;score=\frac{MIC\;(HDP\;in\;combination)}{MIC\;(HDP\;alone)}+\frac{MIC\;(antibiotic\;in\;combination)}{MIC\;(antibiotic\;alone)}$$

A FICI score of ≤0.5 indicates synergism, a FICI score between 0.5 and 1 represents an additive effect, a FICI score between 1 and 4 demonstrates no interaction between the agents, and a FICI score > 4 implies antagonism [28]. For studies reporting multiple FICI scores against a bacterial species (i.e., multiple strains assessed), the mean FICI score was calculated for each species.

### 2.4. Data Synthesis

For the data syntheses, the included articles were stratified according to the kingdom of life of the peptides’ species of origin and grouped accordingly for discussion. Both quantitative and qualitative information were summarised using textual descriptions. A flow diagram of approaches to assessing synergy was generated with SankeyMATIC (version 1.1) [29]. Due to the extensive array of peptides examined here, an appropriate method was required for their categorisation. There are several means of delineating peptides systematically [30], and species of origin and de novo groupings were used due to the lack of uniform information on peptide characteristics.

## 3. Results

### 3.1. Bacterial HDPs

The first AMP identified, gramicidin, was derived from the soil bacterium *Bacillus brevis* [31,32], and its discovery was a first step in identifying a library of now almost innumerable peptides across the kingdoms of life. Ten HDPs and their derivatives from bacterial species were analysed for their interactions with antibiotics against AMR bacteria (Table 1—Kingdom: Bacteria). HDPs produced by prokaryotes (i.e., bacteria and archaea) are unique due to their activity against other bacteria, their fostering of particular microbial communities, and their selection for commensal partners [33].

The HDP p138c, derived from *Bacillus subtilis* from fermented foods, was found to synergise with PC class antibiotics against vancomycin-resistant *S. aureus*, also acting additively against *E. faecium* and *Enterococcus faecalis* [34]. In contrast, its activity was antagonistic with GP and PP classes and indifferent in combination with FQ class antibiotics. Most notably, antagonism was recorded with vancomycin, indicating that p138c does not re-sensitise resistant *S. aureus* to vancomycin.

Laterosporulin10, derived from a soil *Brevibacillus* sp., demonstrated improved activity against *Mycobacterium tuberculosis* when in combination with RF class antibiotics; however, this was not accurately assessed [35].

Durancin 61A, derived from *Enterococcus durans*, was assessed against a panel of Gram-positive and -negative bacteria, revealing indifferent-to-antagonistic interactions with GP and TC class antibiotics; however, synergy was found with vancomycin against vancomycin-intermediate-sensitive *S. aureus*, and an additive interaction was observed with the TC class against *E. faecium* and *Streptococcus* spp. [36]. Durancin 61A also synergised with other HDPs, reuterin and pediocin, against *Clostridium dificile*. L12, derived from a clinical *E. faecium* strain, synergises with FQ and GP classes, acts additively with AG and TC classes, and is indifferent with CS class antibiotics, all against *S. aureus* [37].

Most bacterial HDPs were derived from lactic acid bacterial species (*Lactococcus* and *Pediococcus* spp.), omnipresent in fermentation and agricultural biotechnology. Nisin demonstrates synergy against *Enterococcus* spp. and *S. aureus* in combination with ramoplanin [38]. In combination with AM, it acts additively against *Enterococcus* spp. and antagonistically against *S. aureus*, while interaction with PP class antibiotics was indifferent against *Enterococcus* spp. The nisin Z derivative and Pediocin PA-1/AcH also demonstrated synergistic activity with all classes of antibiotics tested against *Pseudomonas fluorescens* [39]. The lantibiotic derivative, lacticin 3147, demonstrated synergistic activity with PM against *Cronobacter sakazakii* and *Escherichia coli*, additive activity against *E. faecium* and *S. aureus*, indifference against *Bacillus cereus,* and antagonism against the *Salmonella enterica* serotype Typhimurium [40]. The component peptides of lacticin 3147, Ltnα, and Ltnβ were similarly additive-to-synergistic against *E. coli*.

The N-terminal region of the *Helicobacter pylori* ribosomal protein L1, HP (2–20) has improved activity against *P. aeruginosa* in combination with both cefazolin sodium [41] and ciprofloxacin [42] but was not formally assessed. An alternate fragment of the same protein, HP (4–16), has been conjugated to both lactoferricin and plectasin to form the LHP7 hybrid peptide that demonstrated synergistic activity in combination with PC class antibiotics, additive activity with AG, GP, RF, and TC classes, and antagonistic activity with AM classes, all against methicillin-resistant *S. aureus* [43].

Finally, cyclic dipeptides isolated from *Achromobacter* spp. cultured from nematode guts acted synergistically with ampicillin against a range of Gram-positive and -negative planktonic bacteria and bacteria in biofilms [44].

All classes of antibiotics except CP, ML, and PG were assessed in combination with HDPs of bacterial origin. Only one study assessed the activities of HDPs and antibiotics on bacteria in biofilms. Curiously, interactions featuring bacterial HDPs accounted for the most antagonistic interactions recorded. This variety of HDPs sourced solely from bacteria provides a glimpse into the diversity of peptides used by bacteria as both killing agents against unrelated species and inhibitory or regulatory agents of similar species. Other prokaryotic species (i.e., Archaea) may harbour HDPs with paradigm-shifting potential. However, the dissection of these peptides and their specific interactions has only been sparingly explored; their synergies with other HDPs and antibiotics are more so.

### 3.2. Fungal HDPs

Despite a broad array of HDPs identified from fungi, such species are more often used as targets for AMPs than their potential source. Three peptides derived from a single HDP from *Pseudoplectonia nigrella* were identified from the literature (Table 1—Kingdom: Fungi). Plectasin was used as a negative control in combination treatments against *E. coli* [45] and acts additively with PC, RF, and TC classes, indifferently with AG and GP classes, and antagonistic with AM class antibiotics against *S. aureus* [43]. The plectasin derivative NZ2114 demonstrates indifferent-to-synergistic activity in combination with GP, PG, and other classes against *E. faecalis* [46]. The activities of the plectasin-containing LHP7 hybrid peptide are detailed above (see Section 3.1).

All antibiotic classes, except CP, FQ, ML, and PP, were assessed in combination with HDPs of fungal origin. These three examples from a single species demonstrate a sampling of the potent untapped potential of the fungal kingdom to provide versatile HDPs to complement antibiotics against AMR bacteria.

### 3.3. Plant HDPs

Although arguably less studied than other groupings of HDPs, those derived from plants offer not only structural and functional diversity but *in planta* production at scale [47,48]. Two HDPs derived from a plant species were identified from the literature (Table 1—Kingdom: Plantae). IbAMP4, derived from *Impatiens balsamina*, has additive activity with vancomycin and oxacillin against *E. faecalis* but indifferent activity against *K. pneumoniae* in combination with vancomycin. [49]. NuriPep 1653 from *Pisum sativum* is also synergistic with colistin to eliminate *A. baumannii* [50].

The plant kingdom is the least explored for synergistic potential against AMR bacteria and only featured interactions with GP, PC, and PM class antibiotics. While no antagonistic or non-assessed interactions were recorded, extrapolation is difficult due to this limited sample. The activities of these HDPs demonstrate the utility of the plant kingdom to provide novel peptides sourced from such abundant species.

### 3.4. Invertebrate HDPs

Invertebrate HDPs, though largely studied in model organisms such as *Drosophila melanogaster* [51,52], were identified for this review across *Annelida*, *Arthropoda*, and *Xiphosura* phyla. Thirteen HDPs and their derivatives from invertebrate species were identified from the literature (Table 1—Kingdom: Animalia—Phyla Annelida, Arthropoda, and Xiphosura).

Arenicin-1 from *Arenicola marina* synergises with AM, ML, and PC antibiotics against *E. coli*, *S. aureus*, *Staphylococcus epidermidis*, *P. aeruginosa*, and *E. faecium*, the lattermost being an additive instead for PC class antibiotics [53]. Its activity was also established to be due to hyperactivation of the electron transport chain, hydroxyl radical formation, and subsequent depletion of bacterial NADH^+^. Partially contradicting this, another study determined that arenicin-1 was mostly additive or indifferent in combination with the same classes and more against *E. coli*, *P. aeruginosa*, and *S. aureus* [54].

A principal component of bee venom, melittin, synergises against *P. aeruginosa* with doripenem and ceftazidime and against *A. baumannii* with doripenem but is antagonistic with doxycycline and colistin against the latter [55]. A conjugate with vancomycin, hecate, also acts to sensitise otherwise vancomycin-resistant *S. aureus*, but its synergistic potential was not assessed [56]. The melittin derivative, MelitAP-27, acted indifferent-to-synergistic with AM, FQ, ML, PC, and RF class antibiotics against *S. aureus* and *P. aeruginosa* strains [57]. This study further found synergy against bacteria in biofilms for given combinations except AM for *P. aeruginosa*. CM11, an 11-residue derivative of both melittin and cecropin A from the giant silk moth, is indifferent-to-synergistic with AG, CS, FQ, PC, and RF against *A. baumannii*, *E. coli*, *K. pneumoniae*, *P. aeruginosa*, *S. aureus*, and *S.* Typhimurium [58].

A linear analog of thanatin from *Podisus maculiventris* was additive in combination with colistin against *E. coli* [59]. Coprisin from *Scarabaeus satyrus* acts synergistically in combination with GP and PC classes against *E. coli*, *E. faecium*, *S. aureus*, *Streptococcus mutans,* and *P. aeruginosa* but is only additive in combination with CP class antibiotics against *E. coli*, *S. aureus*, and *S. mutans* [60]. These combinations were also tested against the same bacteria in biofilms without assessment of their synergy.

A3, a derivative of AamAP1 from the scorpion *Androctonus amoeruxi*, synergises with AM, FQ, ML, and RF class antibiotics against *S. aureus*, and FQ against *E. faecium*, being additive for other combinations [61]. Hp1404 and BmKn-22, from *Heterometrus petersii* and *Mesobuthus martensii* Karsch, respectively, synergise with kanamycin against *S. aureus* planktonic bacteria and azithromycin against *P. aeruginosa* in biofilms [62,63].

Cecropin A2 from *Aedes aegypti* synergises with tetracycline against *P. aeruginosa*, both in vitro and in vivo, in a wax moth (*Galleria mellonella)* larvae infection model, where the combination resulted in improved host survival compared to either agent individually [64]. The activities of the cecropin A-containing CM11 peptide are detailed above (see Section 3.4). CAMA-syn, derived from both cecropin A and magainin-2, synergises with the synthetic antibacterial compounds 3,6-dihydroxyflavone and YKAs3001 and was additive with naringenin against vancomycin-susceptible and -resistant *E. faecalis* [65].

Anoplin, derived from *Vespula vulgaris* venom, synergises with PM and RF class antibiotics against *E. coli*, *K. pneumoniae*, and *P. aeruginosa* [66]. Variants of anoplin, synthesised as peptide dimers and with fatty-acid (FA) moieties, were developed to extend its native biological and antibacterial activity. Anoplin dimers and dimers of anoplin derivatives are additive in combination with PC against *S. aureus* and RF against *E. coli* [67], whereas FA-anoplin acts indifferent-to-synergistic in combination with AG, GP, PM, and RF classes against *E. coli* and *S. aureus* [68]. FA-anoplin, and its D4,7 dimer, synergise with PM and RF classes against *K. pneumoniae* and *P. aeruginosa* [66]. ZL-2, a peptide inspired by alloferon-1 from *Calliphora vicina*, acts largely synergistically with CS, ML, and PC classes [69]. Transportan-10, a derivative of mastoparan from *Vespula lewisii* and the neuropeptide galanin, has additive activity against *E. faecium*, *E. faecalis*, and *S. aureus* when conjugated to vancomycin [70].

Finally, the activity of tachyplesin III from *Tachypleus gigas* was additive or synergistic with imipenem against *P. aeruginosa*, although whether this interaction was synergistic was not formally assessed [71]. Further, the study investigated the combination against *P. aeruginosa*-induced sepsis in a mouse model, showing significantly better host survival than either agent alone.

The HDPs of the invertebrate phyla demonstrate interactions with all antibiotic classes assessed except the PG class. No antagonistic interactions were recorded for combinations of invertebrate HDPs with antibiotics. Together, these interactions offer a glimpse of the HDP–antibiotic potential within the invertebrate phyla.

### 3.5. Vertebrate HDPs

HDPs have been identified among vertebrates, from marine chordates to terrestrial mammals. Twenty-six HDPs and their derivatives from vertebrate species were identified from the literature (Table 1—Kingdom: Animalia—Phylum Chordata).

Two HDPs isolated from fish, LEAP-2 and pleurocidin-1, are synergistic with PC class antibiotics against *Vibrio harveyi* and *Vibro parahaemolyticus* and AM, ML, and PC class antibiotics against a panel of Gram-positive and -negative pathogens, respectively [72,73]. Other *Vibrio* and *Aeromonas* spp. were also investigated but not assessed appropriately, including in mice and topmouth culter (*Culter alburnus*) in vivo models of *A. hydrophila* infection [73].

Six peptides isolated from amphibians and reptiles were detailed in the literature. Ranalexin, in combination with lysostaphin, facilitates the clearance of methicillin-, vancomycin-, and lysostaphin-resistant *S. aureus*, both *in vitro* and in rabbit, mouse, and cotton rat models of infection [74,75]. Brevinin-2CE acts synergistic-to-additive with AM, FQ, PC, and RF class antibiotics against *S. aureus* and *E. coli* [76]. Temporin G acts in concert with tobramycin against *S. aureus* [77]. Magainin-2 from *Xenopus laevis* has diverse activity itself, but when conjugated to vancomycin, can effectively complement the activities of the two components against methicillin- and vancomycin-resistant *S. aureus* and *Enterococci* spp., but was not assessed formally [78]. The activities of the magainin-2-containing CAMA-syn hybrid peptide are detailed above (see Section 3.4). OH-Cath-30, a cathelicidin derived from king cobra venom, synergises with ciprofloxacin and levofloxacin but is indifferent in combination with gentamicin and cefoperazone against *P. aeruginosa* [79]. While there is much interest and opportunity to exploit the properties of amphibian and reptile HDPs [80,81], investigation of their synergies remains limited.

Cathelicidins are a family of vertebrate HDPs that are crucial in driving innate immunity against pathogens. They are widely distributed in vertebrates, only possessing one variant in humans (LL-37), and are expressed across the skin, respiratory, haematopoietic, and gastrointestinal tissues. Cathelicidins exhibit broad-spectrum antimicrobial activity against bacteria, viruses, and fungi and are also involved in modulating inflammation and wound healing. Further, they enhance the adaptive immune response by promoting the recruitment and activation of immune cells, particularly after stimulation with immunomodulatory agents [82]. LL-37 and its many variants have been developed to enhance its native antimicrobial and immunomodulatory activities while preserving them under physiological change [83]. The activity of LL-37 in combination with antibiotics has been investigated against the most relevant ESKAPE pathogens, showing that its synergy with AG, AM, CS, FQ, GP, PM, and TC class antibiotics against Gram-positive and -negative bacteria is extensive [84,85,86,87,88,89,90,91,92,93,94,95,96,97,98,99,100,101]. An exception was a lack of synergism noted with daptomycin-, linezolid-, and vancomycin-treated *S. aureus* not becoming susceptible to subsequent LL-37 killing [95]. The LL-37 derivatives, LL-13/-17, FK-13-a1 to -a7, FK-16, KR-12-a5, SAAP-148, and SAAP-276 also principally synergise with AM, FQ, GP, PC, and other classes of antibiotics against primarily *P. aeruginosa* and *S. aureus* [88,97,98,99,102,103]. The exceptions are the additive interactions between SAAP-148 and SAAP-276 with teicoplanin against *S. epidermidis* [99].

LL-37 homologues across the chordate phylum include CRAMP from *Mus musculus*, fowlicidin-3 from *Gallus domesticus*, bactenecin and indolicidin from *Bos taurus*, and novicidin from *Ovis aries*. CRAMP conjugated with vancomycin demonstrated robust activity against an array of Gram-positive and -negative bacteria and their biofilm formation [104]. CRAMP also acts additively with nafcillin when employed against *S. aureus* but was not formally assessed [95]. Fowlicidin-3 synergises with AM and PC-class antibiotics against *P. aeruginosa* [100]. Bactenecin and its variants and derivatives, including IDR-1018 and DP7, act additive-to-synergistic across many antibiotic classes against *A. baumannii*, *E. coli*, *K. pneumoniae*, *P. aeruginosa*, *S. aureus*, and *S.* Typhimurium, but with notable indifference against *P. aeruginosa* with AG and CP classes [91,105,106]. This activity is also recapitulated by caprine bactenecin, and the similar protegrin-1 from *Sus domesticus* [93]. Indolicidin and its derivatives act similarly in such combinations [91,107]. The activities of the indolicidin-containing LHP7 hybrid peptide are detailed above (see Section 3.1). LHP7 featured the only antagonistic interaction of the vertebrate HDPs, with AM against *S. aureus*. Novicidin acts additive-to-synergistic with rifampin, ceftriaxone, and ceftazidime against *Enterobacter cloacae*, *E. coli*, and *K. pneumoniae* [45].

Defensins are small peptides that, like LL-37, play a crucial role in vertebrate innate immunity. These HDPs are found principally in neutrophils, mononuclear phagocytes, and epithelial cells [108]. hBD-1 and hBD-3 act additively and synergistically, respectively, against *E. coli* and *S. aureus,* and the latter synergises against *A. baumannii* and *Micrococus luteus*, in combination with rifampicin and amikacin [93,109]. hBD-2 acts additive-to-synergistic against *A. baumannii* and *M. luteus* in combination with AG, FQ, PC, PM, and RF classes. A derivative of hBD-2, hPAB-β, acts in concert with oxacillin against *S. aureus* as a sequential treatment but was not formally assessed [110]. Human neutrophil proteins 1 (hNP-1) and 4 (hNP-4) demonstrate indifferent-to-synergistic activities in combination with AG, FQ, GP, PC, PM, and RF class antibiotics against *A. baumannii, E. coli*, *E. faecium*, *M. luteus*, and *S. aureus*, in studies where synergy was formally assessed [89,93,95,109].

Other human HDPs, i.e., α-MSH, CXCL14, tPMP, thrombocidin-1, and galanin, have been derived to counter the shortcomings of the native peptides. Ana-10, a peptide derived from α-MSH, has indifferent-to-synergistic activity against *S. aureus*, synergising with CS and ML classes [111]. Three derivatives of CXCL14, CXCL14-C17-a1 through a3, are additive-to-synergistic against *P. aeruginosa* in combination with AM and FQ classes while acting additively in combination with PC class antibiotics [101]. tPMP and its derivative RP-1 complement PC against *E. faecium* [89], and the latter acts in concert with PC against *S. aureus* [95]. TC84, a thrombocidin derivative, is indifferent-to-synergistic with teicoplanin against *S. aureus* [99]. The activities of the galanin-containing transportan-10 hybrid peptide are detailed above (see Section 3.4).

The formulation of a mouse ubiquicidin derivative, UBI_29-41_, into quantum dot nanoparticles, in combination with vancomycin and methicillin, expedited its delivery to infection sites in a mouse model but did not amplify their effects against *S. aureus* and *B. subtilis* [112].

Interestingly, despite the extensive array of vertebrate peptides, no interactions between vertebrate HDPs and PG or PP antibiotic classes were documented. This range of vertebrate HDPs demonstrates the diversity of peptides amongst a single phylum. The emphasis of many studies on LL-37, its homologues, and derivatives reflects an anthropocentric focus despite the synergistic potential of other chordate HDPs.

### 3.6. De Novo HDPs

Rationally designed peptides from the literature that were not directly inspired by native HDPs were designated as de novo peptides. Eighteen HDPs developed de novo were identified from the literature (Table 1—De novo). While drawing structural inspiration from native HDPs, such peptides and mimetics are targeted to maximise these activities through conformations otherwise unattainable via natural processes.

The M33 tetra-branched peptide, designed and optimised from an *E. coli* phage library, acts complementary to levofloxacin against *E. coli* and *P. aeruginosa*; however, its conjugation to levofloxacin did not substantially increase the activity of M33 alone [113]. A further study demonstrated that M33’s activity extends to AG, CP, PC, and RF classes against *A. baumannii* and *K. pneumoniae* [114]. UP-5, a pentapeptide composed of only arginine and biphenylalanine, has demonstrated strong activity alone against Gram-positive species while also being synergistic with AM, FQ, and RF classes against *S. aureus*, and *P. aeruginosa* [115]. The M(LLKK)_2_M peptide synergised with rifampicin against—and restored susceptibility to—*Mycobacterium smegmatis*, *Mucobacterium bovis*, and *M. tuberculosis* [116]. ASU014, engineered for binding and inhibition of *S. aureus*, was synergistic in combination with oxacillin and nafcillin against an array of resistant strains [117]. B2088 similarly synergises with AG, AM, and CP antibiotics against *K. pneumoniae* and *P. aeruginosa* [118]. The ARV-1502 peptide synergises with meropenem against *E. coli*, whereas its dimer, A3-APO, synergises with colistin against *A. baumannii* and *K. pneumoniae*, but was only additive in combination with imipenem against *K. pneumoniae* [119]. In the same study, A3-APO improved survival in mouse models of bacteremia with *E. coli* in combination with sub-therapeutic doses of colistin. ARV-1502 acted similarly in a mouse model of melioidosis with *Burkholderia pseudomallei* in combination with ceftazidime. The WLBU2 peptide, composed solely of tryptophan, valine, and arginine residues, complements AG, CP, FQ, and PC classes against *A. baumannii* and *K. pneumoniae* in biofilms [120]. Other rationally designed peptides, HHC-53 and LOP1 through 5, acted additively-to-synergistic against *P. aeruginosa* [91].

D-enantiomeric derivatives of synthetic peptides show promise against planktonic and biofilm-established bacteria, leveraging their established activities with resistance to protease degradation [121]. DJK-5 and -6 act additively to synergistically with AG, CP, CS, and FQ classes against *A. baumannii*, *E. coli*, *K. pneumoniae*, *P. aeruginosa*, and *S.* Typhimurium [122] but demonstrate strain-dependent synergy against planktonic and biofilm-established *K. pneumoniae* [123]. The D-enantiomer of the synthetic DD4 peptide, in combination with colistin, also improves host survival in a *Caenorhabditis elegans* model of both *A. baumannii* and *P. aeruginosa* infection [124]. Several peptidomimetic compounds, accumulated from available arrays, synergise with azithromycin and rifampicin against *E. coli* and *K. pneumoniae* [125].

Conjugating polybasic residues of diaminobutyric acid to levofloxacin also potentiates its activity and enhances its synergy with other FQ-class antibiotics [126]. The lysine-tryptophan dipeptide KW-OBn individually has antibacterial activity against *S. aureus* and is potentiated against *E. coli*, *P. aeruginosa*, and *S. epidermidis* when conjugated to neomycin B [127]. P5 is additive with meropenem against *P. aeruginosa*, though not formally assessed, but demonstrated limited activity against biofilms in combination with gentamycin and tobramycin [128]. Conjugation of kanamycin to the proline-rich peptide P14LRR improved the susceptibility of *A. baumannii*, *E. faecium*, *K. pneumoniae*, *P. aeruginosa*, *S. aureus*, and *S. epidermidis* strains, where kanamycin, tobramycin, and linezolid individually were unable to inhibit these bacteria [129].

Lipidation of HDPs can enhance their delivery and half-life and decrease their immunogenicity [130]. A short lipidated synthetic HDP, BA250-C10, acts synergistically against *P. aeruginosa* combined with colistin and tobramycin [131].

Despite a large group of peptides explored, the de novo group did not explore any activity between peptides and the PG and PP antibiotic classes. Studies of de novo peptides largely featured appropriate synergistic assessment and demonstrated various experimental methods that showcase the utility of their various augmentations in improving HDP properties. 

**Table 1:** Assessments of antibacterial activity between HDPs (and derivatives) and antibiotics. The matrix of peptides and antibiotic classes indicates synergistic (green), additive (blue), indifferent (hashed grey), and antagonistic (orange) interactions between agents against the species noted. Assessments of synergy in biofilms (purple border) or in vivo (orange border) are likewise denoted. Uncolored boxes indicate no assessment metric of synergy used for the interaction. Antibiotic abbreviations: AG, aminoglycosides; AM, amphenicols; CP, carbapenems; CS, cephalosporins; FQ, fluoro/quinolones; GP, glycopeptides; ML, macrolides; PC, penicillins; PG, phosphoglycolipids; PM, polymixins; PP, polypeptides; RF, rifamycins; TC, tetracyclines. *Bacterial abbreviations*: *Ab*, *Acinetobacter baumannii*; *Ah*, *Aeromonas hydrophila*; *As*, *Aeromonas sobria*; *Bc*, *Bacillus cereus*; *Bp*, *Burkholderia pseudomallei*; *Bs*, *Bacillus subtilis*; *Ca*, *Cutibacterium acnes*; *Cd*, *Clostridium dificile*; *Cs*, *Cronobacter sakazakii*; *Ecl*, *Enterobacter cloacae*; *Ec*, *Escherichia coli*; *Efm, Enterococcus faecium*; *Efs*, *Enterococcus faecalis*; *Kp*, *Klebsiella pneumoniae*; *Mb*, *Mycobacterium bovis*; *Mc*, *Moraxella catarrhalis*; *Ml*, *Micrococcus luteus*; *Ms*, *Mycobacterium smegmatis*; *Mt*, *Mycobacterium tuberculosis*; *Pa*, *Pseudomonas aeruginosa*; *Pf*, *Pseudomonas fluorescens*; *Pm*, *Pseudomonas mirabilis*; *Pp*, *Pseudomonas putida*; *Pv*, *Pseudomonas vulgaris*; *Sa*, *Staphylococcus aureus*; *Se*, *Staphylococcus epidermidis*; *Sf*, *Staphylococcus faecalis*; *Sm*, *Streptococcus mutans*; *ST*, *Salmonella enterica* serotype Typhimurium; *Str*, *Streptococcus* spp.; *Va*, *Vibrio anguillarum*; *Vc*, *Vibrio cholerae*; *Vh*, *Vibro harveyi*; *Vp*, *Vibro parahaemolyticus*; *Vs*, *Vibrio splendidus*; *Vv*, *Vibro vulnificus*; *Ye*, *Yersinia enterocolitica*.

**Kingdom:** **Bacteria** *Phylum*	Origin Species	Peptide	Derivative	Antibiotic Class														
				AG	AM	CP	CS	FQ	GP	ML	PC	PG	PM	PP	RF	TC	Oth.	Ref.
*Bacillota*	*Bacillus subtilis*	p138c						*Efm* *Efs* *Sa*	*Efm* *Efs* *Sa*		*Sa*			*Efm* *Efs* *Sa*				[34]
											*Efm* *Efs*							
	*Brevibacillus* sp.	Laterosporulin10													*Mt*			[35]
	*Enterococcus durans*	Durancin 61A							*Sa*							*Efm* *Str*		[36]
									*Cd* *Ec* *Str*							*Cd* *Sa*		
									*Efm*							*Ec*		
	*Enterococcus faecium*	L12		*Sa*			*Sa*	*Sa*	*Sa*							*Sa*		[37]
	*Lactococcus lactis*	Nisin			*Efm* *Efs*									*Efm* *Efs*			*Efm* *Efs* *Sa*	[38]
					*Sa*													
**Kingdom:** **Bacteria** *Phylum*	Origin Species	Peptide	Derivative	Antibiotic Class														
				AG	AM	CP	CS	FQ	GP	ML	PC	PG	PM	PP	RF	TC	Oth.	Ref.
*Bacillota*	*Lactococcus lactis*	Nisin	Nisin Z	*Pf*	*Pf*				*Pf*		*Pf*				*Pf*	*Pf*	*Pf*	[39]
		Lacticin 3147											*Cs* *Ec*					[40]
													*Efm* *Sa*					
													*Bc*					
													*ST*					
			Ltnα/β										*Ec*					
													*Ec*					
	*Pediococcus* spp.	Pediocin PA-1/AcH		*Pf*	*Pf*				*Pf*		*Pf*				*Pf*	*Pf*	*Pf*	[39]
*Campylobacterota*	*Helicobacter pylori*	HP (2–20)					*Pa*	*Pa*										[41,42]
		HP (4–16)	LHP7	*Sa*	*Sa*				*Sa*		*Sa*				*Sa*	*Sa*		[43]
*Pseudomonadota*	*Achromobacter* spp.	Cyclic Dipeptides									*Bs* *Efm* *Kp* *Pa Pm* *Pv* *Sa* *Se* *Sf* *ST*							[44]
											*Bs* *Efm* *Kp* *Pa Pm* *Pv* *Sa* *Se* *Sf* *ST*							
**Kingdom:** **Fungi** *Division*	Origin Species	Peptide	Derivative	Antibiotic Class														
				AG	AM	CP	CS	FQ	GP	ML	PC	PG	PM	PP	RF	TC	Oth.	Ref.
*Ascomycota*	*Pseudoplectania nigrella*	Plectasin					*Ec*								*Ec*			[45]
				*Sa*	*Sa*				*Sa*		*Sa*				*Sa*	*Sa*		[43]
			LHP7	*Sa*	*Sa*				*Sa*		*Sa*				*Sa*	*Sa*		
			Plectasin NZ2114						*Efs*			*Efs*					*Efs*	[46]
									*Efs*			*Efs*						
**Plantae** *Order*																		
*Ericales*	*Impatiens balsamina*	Ib-AMP4							*Efs*		*Efs*							[49]
									*Kp*									
*Fabales*	*Pisum sativum*	NuriPep 1653											*Ab*					[50]
**Animalia** *Phylum* Order																		
*Annelida* Sedentaria	*Arenicola marina*	Arenicin-1			*Ec* *Efm* *Pa* *Sa* *Se*					*Ec* *Efm* *Pa* *Sa* *Se*	*Ec* *Pa* *Sa* *Se*							[53]
											*Efm*							
				*Ec* *Pa* *Sa*	*Ec* *Pa* *Sa*				*Pa* *Sa*	*Sa*	*Ec* *Pa* *Sa*		*Ec* *Pa* *Sa*		*Ec* *Sa*	*Ec*	*Ec* *Pa* *Sa*	[54]
										*Ec* *Pa*						*Pa* *Sa*		
									*Ec*						*Pa*			
*Arthropoda* Hymenoptera	*Apis mellifera*	Melittin				*Ab Pa*	*Pa*						*Ab*			*Ab*		[55]
			Hecate						*Sa*									[56]
			MelitAP-27		*Pa*			*Sa*		*Pa* *Sa*	*Sa*				*Sa*			[57]
					*Sa*			*Pa*							*Pa*			
					*Pa*			*Pa* *Sa*		*Sa*					*Sa*			
**Kingdom:** **Animalia** *Phylum* Order	Origin Species	Peptide	Derivative	Antibiotic Class														
				AG	AM	CP	CS	FQ	GP	ML	PC	PG	PM	PP	RF	TC	Oth.	Ref.
*Arthropoda* Hymenoptera	*Apis mellifera*	Melittin	CM11	*Ab* *Ec* *Sa*			*Pa*	*Ab* *Kp*			*Ec* *Sa*				*Sa*			[58]
				*Kp* *Pa*			*Ab* *Ec* *Kp* *ST*	*Ab* *Ec* *Kp* *Pa* *Sa*			*Ab* *Kp* *ST*							
Hemiptera	*Podisus maculiventris*	Linear Thanatin											*Ec*					[59]
Coleoptera	*Copris tripartitus*	Coprisin			*Efm* *Pa*				*Ec* *Efm* *Pa* *Sa* *Sm*		*Ec* *Efm* *Pa* *Sa* *Sm*							[60]
					*Ec* *Sa* *Sm*													
					*Ec* *Efm* *Pa* *Sa* *Sm*				*Ec* *Efm* *Pa* *Sa* *Sm*		*Ec* *Efm* *Pa* *Sa* *Sm*							
Arachnida	*Androctonus amoeruxi*	AamAP1	A3		*Sa*			*Efm Sa*		*Sa*					*Sa*			[61]
					*Efm* *Sa*			*Sa*		*Efm* *Sa*					*Efm* *Sa*			
	*Heterometrus petersii*	Hp1404		*Sa*														[62]
	*Mesobuthus martensii* Karsch	BmKn-22								*Pa*								[63]
Insecta	*Hyalophora cecropia*	Cecropin A	CAMA-syn														*Efs*	[65]
																	*Efs*	
			CM11	*Ab* *Ec* *Sa*			*Pa*	*Ab* *Kp*			*Ec* *Sa*				*Sa*			[58]
				*Kp* *Pa*			*Ab* *Ec* *Kp* *ST*	*Ab* *Ec* *Kp* *Pa* *Sa*			*Ab* *Kp* *ST*							
**Kingdom:** **Animalia** *Phylum* Order	Origin Species	Peptide	Derivative	Antibiotic Class														
				AG	AM	CP	CS	FQ	GP	ML	PC	PG	PM	PP	RF	TC	Oth.	Ref.
*Arthropoda* Insecta	*Aedes aegypti*	Cecropin A2														*Pa*		[64]
																*Pa*		
	*Anoplius samariensis*	Anoplin											*Ec Kp Pa*		*Ec Kp Pa*			[66]
			Anoplin/-RW Dimers								*Sa*				*Ec*			[67]
			FA-anoplin	*Ec*					*Ec* *Sa*				*Ec* *Sa*		*Sa*			[68]
				*Sa*											*Ec*			
													*Ec* *Kp* *Pa*		*Ec* *Kp* *Pa*			[66]
													*Pa*					
			FA-anoplin Dimers										*Ec Kp Pa*		*Ec Kp Pa*			
													*Pa*					
	*Calliphora vicina*	Alloferon-1	ZL-2				*Ec Kp Pa Sa Se*			*Ec Pa* *Se*	*Ec Kp Pa Sa Se*							[69]
										*Kp* *Sa*								
	*Vespula lewisii*	Mastoparan	Transportan-10						*Efm Efs Sa*									[70]
Xiphosura	*Tachypleus gigas*	Tachyplesin III				*Pa*												[71]
**Kingdom:** **Animalia** *Phylum* Order	Origin Species	Peptide	Derivative	Antibiotic Class														
				AG	AM	CP	CS	FQ	GP	ML	PC	PG	PM	PP	RF	TC	Oth.	Ref.
*Chordata* Cypriniformes	*Culter alburnus*	LEAP-2									*Vh* *Vp*							[73]
											*As Ah* *Va* *Vc* *Vh Vp Vv* *Vs*							
											*Ah*							
Pleuronectiformes	*Pleuronectes americanus*	Pleurocidin-1			*Ca Ec Pa* *Sa*					*Ca Ec Efm Pa* *Sa*	*Ca Ec Efm* *Pa Sa*							[72]
					*Efm*													
Amphibia	*Rana catesbeiana*	Ranalexin															*Sa* *Se*	[74]
																	*Sa*	
																	*Sa*	[75]
	*Rana chensinensis*	Brevinin-2CE			*Ec Sa*			*Ec Sa*			*Sa*				*Ec*		*Ec*	[76]
															*Sa*		*Sa*	
	*Rana temporaria*	Temporin G		*Sa*														[77]
	*Xenopus laevis*	Magainin 2							*Efm Efs* *Sa* *Mc*									[78]
			CAMA-syn														*Efs*	[65]
																	*Efs*	
Squamata	*Ophiophagus hannah*	OH-CATH30		*Pa*			*Pa*	*Pa*										[79]
								*Pa*										
Galliformes	*Gallus domesticus*	Fowlicidin-3			*Pa*						*Pa*							[100]
**Kingdom:** **Animalia** *Phylum* Order	Origin Species	Peptide	Derivative	Antibiotic Class														
				AG	AM	CP	CS	FQ	GP	ML	PC	PG	PM	PP	RF	TC	Oth.	Ref.
*Chordata* Artiodactyla	*Bos taurus*	Bactenecin		*Pa*		*Pa*	*Pa*	*Pa*		*Pa*	*Pa*		*Pa*			*Pa*		[91]
								*Pa*		*Pa*								
			Variants	*Pa*		*Pa*	*Pa*	*Pa*		*Pa*	*Pa*		*Pa*			*Pa*		
				*Pa*						*Pa*			*Pa*					
			IDR-1018	*Ab* *Ec* *Kp* *Pa*		*Pa* *Sa*	*Ab* *Pa* *Sa* *ST*	*Pa* *Sa*										[105]
						*Ab* *Ec* *Kp* *ST*		*Ac* *Ec* *Kp* *ST*										
				*Sa* *ST*			*Ec* *Kp*	*Pa*										
				*Ec* *Kp*			*Ab* *Sa* *ST*											
			DP7	*Sa*					*Ab* *Ec* *Pa* *Sa*	*Pa* *Sa*	*Ab* *Ec* *Sa*						*Ab* *Pa* *Sa*	[106]
				*Ab* *Ec* *Pa*														
										*Ab* *Ec*								
											*Pa*						*Ec*	
		Indolicidin		*Pa*		*Pa*	*Pa*	*Pa*		*Pa*	*Pa*		*Pa*			*Pa*		[91]
				*Pa*														
			Variants	*Pa*		*Pa*	*Pa*	*Pa*		*Pa*	*Pa*		*Pa*			*Pa*		
										*Pa*								
			Omiganan	*Ec* *Sa*					*Sa*	*Sa*	*Ab*						*Ab* *Pa* *Sa*	[107]
									*Ab* *Pa*	*Ab* *Ec* *Pa*	*Ec*							
				*Ab* *Pa*							*Pa*							
									*Ec*		*Sa*						*Ec*	
			LHP7	*Sa*	*Sa*				*Sa*		*Sa*				*Sa*	*Sa*		[43]
	*Ovis aries*	Novicidin					*Ec* *Ecl* *Kp*								*Ec* *Ecl* *Kp*			[45]	
							*Ec* *Ecl* *Kp*								*Ec* *Ecl* *Kp*			
**Kingdom:** **Animalia** *Phylum* Order	Origin Species	Peptide	Derivative	Antibiotic Class														
				AG	AM	CP	CS	FQ	GP	ML	PC	PG	PM	PP	RF	TC	Oth.	Ref.
*Chordata* Artiodactyla	*Capra hircus*	Bactenecin		*Ab* *Kp* *Pa* *Sa*		*Ab* *Sa*		*Ab* *Ml* *Sa*		*Ec* *Kp* *Pa* *Sa*	*Sa*		*Ab* *Ec* *Ml* *Sa*		*Ec*			[93]
											*Ab* *Ml* *Sa*							
				*Ab* *Ec* *Kp* *Ml* *Sa*		*Ec* *Kp* *Pa*		*Ab* *Ec* *Kp* *Pa* *Sa*			*Ab* *Ec* *Kp* *Pa*				*Ab* *Ml* *Sa*			
										*Ab*								
	*Sus domesticus*	Protegrin-1		*Ab* *Ec* *Kp* *Ml* *Pa*		*Ab* *Sa*		*Kp*		*Kp* *Pa*	*Ab* *Ml* *Sa*		*Ab* *Ec* *Ml* *Sa*		*Ec* *Ml* *Sa*			
								*Ab* *Ec* *Ml* *Sa*										
				*Sa*		*Ec* *Kp* *Pa*		*Pa*		*Ab* *Ec* *Sa*	*Ec* *Kp* *Pa* *Sa*				*Ab*			
										*Ab*								
Rodentia	*Mus musculus*	CRAMP							*Bc Bs Ec Ml Pp Sa ST Ye*									[104]
									*ST*									
											*Sa*							[95]
		Ubiquicidin	ZnO@ PEP-MPA						*Bs* *Ec* *Sa*		*Sa*							[112]
									*Sa*									
**Kingdom:** **Animalia** *Phylum* Order	Origin Species	Peptide	Derivative	Antibiotic Class														
				AG	AM	CP	CS	FQ	GP	ML	PC	PG	PM	PP	RF	TC	Oth.	Ref.
*Chordata* Primates	*Homo sapiens*	Cathelicidin								*Ab* *Kp* *Pa*								[84]
							*Efs*				*Efs*							[85]
							*Sa*											[86]
						*Efm* *Efs*	*Efm* *Efs*				*Efm* *Efs*							[87]
									*Sa*									[88]
											*Efm*							[89]
							*Sa*		*Sa*		*Sa*						*Sa*	[95]
				*Pa Sa Str*														[90]
					*Pa*			*Pa*			*Pa*							[98]
									*Sa*									[99]
									*Se*									
									*Sa* *Se*									
									*Sa*									
					*Pa*						*Pa*							[100]
					*Pa*			*Pa*			*Pa*							[101]
					*Pa* *Sa*													[97]
					*ST*		*ST*	*ST*		*ST*								[92]
									*Sa*									[94]
									*Sa*									
**Kingdom:** **Animalia** *Phylum* Order	Origin Species	Peptide	Derivative	Antibiotic Class														
				AG	AM	CP	CS	FQ	GP	ML	PC	PG	PM	PP	RF	TC	Oth.	Ref.
*Chordata* Primates	*Homo sapiens*	Cathelicidin		*Ec*				*Ec*			*Ec*		*Ec* *Pa*			*Ec* *Pa*	*Ec*	[96]
													*Pa*					
				*Pa*				*Pa*			*Pa*		*Ec* *Pa*				*Pa*	
													*Pa*					
				*Pa*		*Pa*	*Pa*	*Pa*		*Pa*	*Pa*		*Pa*			*Pa*		[91]
				*Ml*				*Ab* *Ml*			*Ml*		*Ab* *Ec* *Ml*		*Ec* *Ml*			[93]
				*Ab* *Ec*				*Ec*			*Ab*				*Ab*			
			LL-13/-17						*Sa*									[88]
									*Sa*									
			FK-13-a1/-a7		*Pa* *Sa*													[97]
			FK-16						*Pa*									[103]
			KR-12-a5		*Pa*			*Pa*			*Pa*							[98]
			SAAP-148														*Sa*	[102]
																	*Ab* *Ec*	
																	*Ec* *Sa*	
				*Sa*			*Sa*	*Sa*	*Sa*						*Sa*	*Sa*	*Sa*	[99]
									*Sa* *Se*									
									*Sa*								*Sa*	
									*Sa*									
			SAAP-276						*Sa*									
									*Sa*									
									*Sa*									
**Kingdom:** **Animalia** *Phylum* Order	Origin Species	Peptide	Derivative	Antibiotic Class														
				AG	AM	CP	CS	FQ	GP	ML	PC	PG	PM	PP	RF	TC	Oth.	Ref.
*Chordata* Primates	*Homo sapiens*	α-MSH	Ana-10	*Sa*			*Sa*	*Sa*	*Sa*		*Sa*				*Sa*	*Sa*	*Sa*	[111]
																	*Sa*		
							*Sa*		*Sa*		*Sa*						*Sa*	
																	*Sa*	
		CXCL14	CXCL14-C17 a1-3		*Pa*			*Pa*			*Pa*							[101]
					*Pa*			*Pa*			*Pa*							
		tPMP									*Efm*							[89]
			RP-1								*Efm*							
									*Sa*		*Sa*							[95]
		hNP-1									*Efm*							[89]
									*Sa*		*Sa*							[95]
				*Ec*											*Sa*			[109]
				*Ml*				*Ab* *Ml* *Sa*			*Ab* *Ml* *Sa*		*Ab* *Ec* *Ml*		*Sa*			[93]
				*Ab* *Ec* *Sa*				*Ec*					*Sa*		*Ab* *Ec* *Ml*			
		hNP-4		*Ab* *Ml* *Sa*				*Ab* *Ml* *Sa*			*Ab* *Ml*		*Ab* *Ml*		*Ml* *Sa*			
											*Sa*		*Sa*		*Ab*			
		hBD-1		*Ec*											*Sa*			[109]
		hBD-2		*Ml*				*Ab* *Ml*			*Ab* *Ml*		*Ab* *Ml*		*Ab*			[93]
				*Ab*											*Ml*				
			hPAB-beta		*Sa*				*Sa*		*Sa*							[110]
**Kingdom:** **Animalia** *Phylum* Order	Origin Species	Peptide	Derivative	Antibiotic Class														
				AG	AM	CP	CS	FQ	GP	ML	PC	PG	PM	PP	RF	TC	Oth.	Ref.
*Chordata* Primates	*Homo sapiens*	hBD-3		*Ec*											*Sa*			[109]
				*Ab* *Ec* *Ml*				*Ec* *Ml* *Sa*			*Ab* *Ml* *Sa*		*Ab* *Ec* *Ml* *Sa*		*Sa*			[93]
															*Ec* *Ml*			
				*Sa*				*Ab*							*Ab*			
		Thrombocidin-1	TC84						*Sa*									[99]
									*Sa*										
									*Sa*									
		Galanin	Transportan-10						*Efm Efs Sa*									[70]
De novo *Class*																		
*Peptides*		M33						*Ec Pa*										[113]
				*Ab* *Kp*		*Ab Kp Pa*		*Ab Kp Pa*							*Ab* *Kp*		*Ab*	[114]
																	*Pa*	
				*Pa*											*Pa*		*Kp*	
		UP-5			*Pa*			*Sa*		*Pa*	*Pa* *Sa*				*Pa*			[115]
					*Sa*			*Pa*		*Sa*					*Sa*			
		M(LLKK)2M													*Mb Ms*			[116]
															*Mt*			
		ASU014									*Sa*							[117]
											*Sa*							
		B2088		*Kp Pa*	*Kp Pa*	*Pa*		*Kp* *Pa*		*Pa*	*Pa*							[118]
				*Pa*		*Pa*		*Pa*										
		ARV1502				*Ec*	*Bp*											[119]
			A3-APO			*Ab*							*Kp*					
						*Kp*												
						*Kp*							*Kp*					
**De novo** *Class*		Peptide	Derivative	Antibiotic Class														
				AG	AM	CP	CS	FQ	GP	ML	PC	PG	PM	PP	RF	TC	Oth.	Ref.
*Peptides*		WLBU2		*Ab Kp*		*Ab Kp*		*Ab Kp*			*Ab Kp*							[120]
		HHC-53		*Pa*		*Pa*	*Pa*	*Pa*		*Pa*	*Pa*		*Pa*			*Pa*		[91]
													*Pa*					
		LOP1-5		*Pa*		*Pa*	*Pa*	*Pa*		*Pa*	*Pa*		*Pa*			*Pa*		
				*Pa*						*Pa*			*Pa*					
*D-enantiomers*		DJK-5/-6		*Ab Ec Kp Pa ST*		*Ab Ec Kp Pa ST*	*Ec Pa*	*Pa*										[122]
							*Ab Kp ST*	*Ab Ec Kp ST*										
				*Ab* *Kp*		*Ab*	*Ec*	*Pa* *ST*										
						*Kp*	*Kp*						*Kp*					[123]
							*Kp*											
						*Kp*	*Kp*						*Kp*					
							*Kp*											
		D-RR4											*Ab Pa*					[124]
		Peptidomimetic							*Ec Kp* *Pa*					*Ec Kp* *Pa*		*Kp*		[125]
*Antibiotic conjugates*		Polybasic Peptide-Levofloxacin					*Ec Kp Pa*											[126]
		kW-OBn		*Ec Pa Sa Se*														[127]
**De novo** *Class*		Peptide	Derivative	Antibiotic Class															
				AG	AM	CP	CS	FQ	GP	ML	PC	PG	PM	PP	RF	TC	Oth.	Ref.	
*Antibiotic conjugates*		P5									*Pa*							[128]
		P14LRR		*Ab Efm Kp Pa Sa Se*														[129]
*Lipidated peptides*		BA250-C10		*Pa*									*Pa*					[131]
				*Pa*									*Pa*					

### 3.7. Summary of Studies into Combinatory Activities of HDPs and Antibiotics against AMR Bacteria

**Figure 2:**
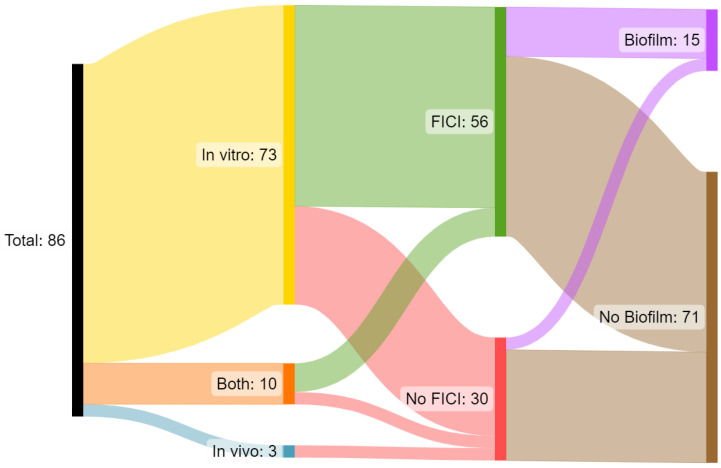
Flow diagram of experimental modeling from the identified studies. Studies were conducted strictly through in vitro models (yellow), in vivo models (blue), or a combination of both (orange). Studies accurately assessed the potential synergies between HDPs and antibiotics through the use of a FICI score (green) or via alternate methods (red) and tested combinations against biofilm-established bacteria (purple) or not (brown). Diagram created using SankeyMATIC [16].

From the compiled studies, a total of 86 met the criteria of our investigation (Figure 2). Seventy-three (84.9%) of these studies assessed the interactions between agents through in vitro experiments only, while ten studies (11.6%) investigated combined activities in both in vitro and in vivo modeling, and only three studies (3.5%) used in vivo models exclusively (i.e., animal models of infection). Only one study involved using host tissue models for infection [102].

FICI scores—derived from checkerboard assay experiments—are integral to determining if the antibacterial activity of agents together is greater than the sum of their activities, based on reducing agents’ effective concentrations between individual and combined applications. From the studies compiled, 56 (65.1%) demonstrated appropriate FICI scores for HDP and antibiotic combinations or appropriate metric derivations, and 30 (34.9%) otherwise implied synergy without an appropriate metric.

Biofilm-encased bacteria are often not included in the assessment of antibiotic candidates, a factor recapitulated among the identified studies, in which only 16 (17.4%) reported activity against bacteria in biofilms, and 73 (82.6%) did not. Predictably, none of the identified in vivo studies assessed synergy with a FICI score.

Interactions between HDPs and antibiotics were reported in 22 Gram-negative and 15 Gram-positive species (Table 2). The most reported species were *P. aeruginosa* (23.1%) and *E. coli* (13.2%) of the Gram-negative grouping, and *S. aureus* (24.3%) and *E. faecium* (4.1%) of the Gram-positive grouping. Aside from those noted, all other members of the ESKAPE panel were reported, constituting *K. pneumoniae* (8.3%), *A. baumannii* (9.6%), and *Enterobacter* spp. (0.4%). Together, the ESKAPE pathogens accounted for 70.0% of the interactions documented.

Additive relationships between HDPs and antibiotics were the largest proportion of interactions, accounting for 39.4% of records noted, whereas synergistic, indifferent, and antagonistic interactions accounted for 32.3%, 11.3%, and 1.5%, respectively. Interactions not assessed via FICI scores accounted for 15.5% of all records. In most interactions documented per bacterial species, synergistic and additive interactions account for the largest proportion of records.

Of the antibiotic classes documented as interacting with HDPs, the penicillin class was the most represented (16.7%), while aminoglycosides (12.6%), fluoroquinolones (11.6%), and glycopeptides (9.7%) all constituted large proportions of antibiotics used in synergistic assessment (Table 3). Synergy was most often reported between HDPs and the PG, ML, and AM classes, accounting for 50.0%, 49.2%, and 41.8% of their respective reported interactions. Notably, antagonism between HDPs and PP and AM classes was more frequently reported for these combinations, i.e., 60.0% and 9.1% of their respective reported interactions.

Together, these trends in the literature indicate that while the appropriate ratification of antimicrobial synergy between HDPs and antibiotics has gained popularity, the advancement of studies to more robust modelling (i.e., advanced models of host tissue and systems, in vivo studies, biofilm assessment), and the involvement of unconventional antibiotics and pathogens lags behind.

## 4. Discussion

### 4.1. Prokaryote HDPs

The prokaryote repertoire of HDPs was largely derived from clinical isolates and bacteria used in fermentation processes. Combinations of these HDPs and their derivatives were investigated with all except CP and PG classes of antibiotics. While this draws attention to the necessity for broader cross-validation, these classes are largely neglected among other studies reviewed here, and resistance to such classes is less noted than others, particularly among ESKAPE pathogens.

Interestingly, the bacterial HDPs featured the largest proportion of antagonistic interactions. This could infer that bacterial HDPs are limited in their capacity to synergise with antibiotics compared to others, particularly against AMR bacteria. However, this is largely anecdotal and more comprehensive analysis of the limitations of bacterial HDPs is required to make concrete conclusions.

While these studies focused on the use of HDPs in synergistic activity against pathogens, there is growing evidence of the utility of such peptides, or indeed their respective bacteria, in addressing dysbiosis and related inflammatory disorders by targeting pathogenic strains while cultivating commensal species [132,133]. The effect of HDPs in combination with antibiotics on commensal species remains minimally explored. 

### 4.2. HDPs from Plants and Fungi

Some of the most robust HDPs can be sourced from plants and fungi, with the foundational antibiotic discovery of penicillin by Alexander Fleming being attributed to a simple bread mould. The limited plant and fungal suite of HDPs were derived from singular species, reflecting the lack of research focus on these kingdoms concerning synergy against AMR bacteria. These HDPs and derivatives were not assessed against AM, CP, CS, FQ, ML, or PM classes of antibiotics. As such, further attempts to identify plant and fungal HDPs for antibiotic synergy should broaden the scope of HDP–antibiotic combinations assessed. However, these HDP groupings were the only ones to assess combinations with the PG class of antibiotics. The production of HDPs via these species, coupled with their ease of culturing and potential for genetic engineering, also offers an avenue for expedient production of these resourceful peptides at a commercial scale and bolstering agricultural stock against spoilage [134,135].

### 4.3. HDPs from Invertebrates

HDPs derived from invertebrate species offer a suite of potent native HDPs and an extensive array of derived peptides and conjugates, originating from lugworms to horseshoe crabs. These HDPs and derivatives were assessed against all except the PG and PP classes of antibiotics. These HDP interactions with antibiotics were largely reported as synergistic; however, no clear trends within the literature regarding antibiotic class or bacteria were identified. The augmentation of peptides and their assessment across planktonic, biofilm, and in vivo models also demonstrated the breadth and adaptability of HDPs from the invertebrate phyla.

### 4.4. HDPs from Vertebrates

Vertebrate HDPs, comprising peptides from fish to humans, were the most studied among the literature reviewed here. Similar to the invertebrate HDPs reviewed, the HDP and derivative interactions here also neglected the PG class of antibiotics, though they provided a spectrum of interaction across all other classes and against a broad range of bacterial species. However, no clear trends can be discerned from the range of interactions assessed among vertebrate HDPs.

Most records expectedly analysed interactions between human cathelicidin or defensins and antibiotics. While these records primarily featured synergistic interactions and featured both Gram-positive and -negative strains, *S. aureus* was the focus of most studies. Only one antagonistic interaction was noted from LHP7, a peptide shared with the bacterial and fungal HDP groupings. Given the extensive research focus on vertebrate HDP–antibiotic combinations, more antagonistic interactions would be expected, if only by chance. This may reflect the bias of positive reporting in the scientific literature, as antagonistic interactions could be viewed negatively and omitted from research publication.

### 4.5. De Novo HDPs

HDPs designed and synthesised via rational design or library mining comprised the second-largest selection of peptide interactions recorded, encompassing branched peptides, enantiomeric analogues, antibiotic conjugates, and lipopeptides. The only peptide interactions with antibiotics neglected were with the CP, PG, and PP classes.

Progress in the rational design and development of peptide therapeutics is reflected by the prominence of de novo peptides reviewed here and their success in synergising with the arrays of antibiotics assessed. This supports rational and iterative augmentation of peptides to diversify and bolster our current antibiotic panels. Commercial development of peptides for antibiotic purposes remains a guarded process, with peptide libraries and leading compounds reserved from public view, lending notable bias in their reporting. It should be noted that many of the alterations and augmentations used integrally in the design of de novo peptides from these studies can similarly be used to develop native HDPs and derivatives further to circumvent their shortcomings (i.e., lipidation and antibiotic conjugation). The discovery process for novel therapeutics from native HDPs is largely either anecdotal, as in the discovery of penicillin, or out of opportunism and subjective selection of species and tissues. Only recently, with the advent of advanced computational modeling, can we rationally design, optimise, validate, and repurpose potential antimicrobial peptides with less biased methodology.

Comprehensive knowledge of true HDP diversity across the kingdoms of life is currently unattainable. As such, while de novo synthesis and augmentation hold much promise in combatting the tide of AMR, it is in our best interest to identify and validate new potential antimicrobial agents from our continually growing resources and bridge limitations using such technology. 

### 4.6. Summary of Studies into Combinatory Activities of HDPs and Antibiotics against AMR Bacteria

From the literature compiled, it is clear—and expected—that *in vitro* assessment of synergy was the prevailing method of investigation, with studies largely investigating agents in combination using checkerboard assays. In vivo studies did not typically feature accurate synergistic assessment, which is also expected, given the limitations of progressing studies in animal models, such as the associated costs and ethical implications of replicating checkerboard assay sample sizes.

Synergism between HDPs and antibiotics was reported accurately by most studies. The studies that did not feature FICI (or similar) scores of agents in combination claimed synergy despite lacking this metric, though typically used another valid but not comparable method. Such studies typically reported the combined use of agents and demonstrated an antibacterial effect greater than either agent individually; however, this does not necessarily constitute a greater effect than the sum of the agents’ individual activities. The latest of these studies was published in 2020, and as such, this does not appear to be an artefact of studies published prior to the recent emphasis on synergistic activity to combat AMR bacteria. Notably, studies that further assessed synergy against bacteria residing in biofilms were the minority, constituted by a distinct proportion of the same studies that did not assess synergy appropriately. No in vivo studies used an appropriate synergy assessment due to the lack of standardised metrics for such analysis. As such, the outcome of enhanced survival of treatment groups with agents in combination over agents individually is typically used as a deciding factor of synergy in these studies. This severely limits our capacity to determine the extent of synergy between agents in vivo. The optimisation of therapy through potentiating sub-optimal doses of either agent, and the mitigation of potential side effects such as cytotoxicity or adverse inflammatory reactions are further limited.

Together, it is clear that although much attention has been paid recently to the appropriate measurement of synergism between agents, much work must be done to expedite the preclinical advancement of promising synergistic HDP therapies. FICI scoring of agents in combination offers the most accurate assessment of HDP–antibiotic synergy in vitro without relying on subjective concentration thresholds. Further, assessing agents’ activities against other bacterial life cycle stages (i.e., biofilms and persisters) is clinically relevant (e.g., infections due to medical instruments and implants) but remains poorly explored throughout the literature. More so, of the few studies that assessed activities against biofilms, only four select studies performed a checkerboard assessment of such activity, yielding appropriate FICI scores [57,105,122,123]. Proper assessment of the synergistic nature of HDP–antibiotic combinations is vital in advancing synergistic combinations against chronic and deep-seated infections, particularly those of AMR bacteria. Although vital to the clinical development of HDP–antibiotic combinations, such studies in animal infection models are scarce. Of note, the animal models used are typically mouse or rabbit, whereas invertebrate *D. melanogaster* and *C. elegans* models, among other species, are fast becoming relevant infection models for rapid in vivo advancement [136,137]. Moreover, we would like to stress that advanced human in vitro models that replicate essential features of the host tissue may also bridge the gap between in vitro and in vivo models while contributing to reducing animal use for research. In this connection, it should be noted that only a single study utilised an advanced tissue infection model to assess synergistic activity between HDPs and an antibiotic agent [102].

From the studies retrieved, synergies between HDPs and antibiotics were reported across both Gram-negative and Gram-positive bacterial groupings, and all species constituting the ESKAPE pathogen panel were reported, constituting the majority of interactions. This was expected after selecting studies featuring AMR strains, as frequent resistance emergence is one of the largest factors attributing to the ESKAPE panel designation. Promisingly, less clinically relevant species were also featured in the literature, if not prominently (e.g., *Cutibacterium acnes* and *P. fluorescens*). Broadening the focus of synergistic investigations is necessary in anticipation of future AMR development and increasing immunocompromised populations.

Synergistic and additive activities being the most reported of the interactions is unsurprising, given both the bias of the literature search conducted and the inherent publication bias of negative results. In many cases, either through the confusion surrounding appropriate metrics or attempts to demonstrate effects in the absence of such, a sizeable portion of the studies noted here presented often aberrant or misleading results. An obvious caveat to this is the assessment of HDP–antibiotic combinations in vivo, as the reporting of synergy via a checkerboard methodology, would, in most cases, be prohibitively expensive, labor-intensive, and ethically compromising in the use of larger animal models (i.e., vertebrates).

The use of penicillins was the most frequently reported, not least due to their abundance and conventional use, but also the pathogen targeted by the combination treatments. Penicillins were most targeted against *S. aureus*, although their overrepresentation within the literature extends beyond just this species. Notably, no selection of HDPs (i.e., from a particular kingdom or phyla) investigated interactions across the complete panel of antibiotic classes. The balance of other antibiotic classes otherwise investigated was far from equal, with the rarer and less clinically conventional classes being similarly less investigated. Many of these rarer classes are considered last-resort antibiotics, so their access is likewise not as uniform across clinical settings. While the focus should be extended to a definitive panel for assessing synergy across all major antibiotic classes, the investigation of synergy is not only to expedite HDP advancement but also to repurpose and restore the utility of antibiotics stifled by the rise of AMR.

## 5. Conclusions

Here, we have provided an overview and discussion of the extensive literature on synergistic antimicrobial activities of combinations of HDPs or their derivatives and established antibiotics. From this, it is clear that PC-class antibiotics are the most used in these studies, while the PG class and other emerging agents remain somewhat understudied. Moreover, most HDPs investigated were of vertebrate or de novo origin. As studies of HDP–antibiotic combinations were not comprehensive in their analysis, it is hard to identify addressable gaps within the literature to be prioritised, other than the noted need for standardization of experimental methods and approaches to assessing synergy and the advancement of in vitro findings against planktonic bacteria to other bacterial life stages and in vivo models. Most of these studies adequately described the synergism of action between agents using the conventional or adapted FICI metric [28]. Seven of the HDPs investigated in this review have advanced to clinical trials at the time of publication (Table 4), demonstrating the tapering development pipeline and the promise of clinical utility for HDPs. It should be noted that while this review focuses on applying HDPs against AMR bacteria, their utility against other potential pathogens, i.e., viruses and fungi, is less explored and a vital opportunity.

Most of the peptides examined here were native peptides or constructs thereof, otherwise without augmentation or advanced formulation. HDP–antibiotic conjugates were rarely additive-to-synergistic against the relevant pathogen, with conjugates usually only matching the activity of equivalent doses of free HDPs and antibiotics. It has been examined previously that by conjugating such agents together, pore-formation, lysis, and targeting intracellular components are limited: the principal activities of the peptides and antibiotics, respectively. This could be mitigated, however, as muted resistance development and/or reduced cytotoxicity from such conjugates are also desired therapeutic traits. It should also be noted that while efficacy and synergism are ideal traits from a combined therapy or conjugate, the wider consequences of antibiotic use, including microbiome depletion and environmental accumulation, are also problems that can be addressed in the formulation of complementary HDP therapeutics via antibiotic sparing.

Despite the challenges, the combined use of HDPs and antibiotics holds promise as a new clinical direction. This approach may expedite HDP-related drug development, expanding therapeutic options against AMR infections and providing insight into the underlying mechanisms of infection and inflammatory response. As researchers navigate these complexities and address the complications outlined, a future marked by effective combination therapies to combat AMR is emerging.

## Figures and Tables

**Table 2:** Reported bacterial species and their combinatory HDP–antibiotic activity totals. Sub-tables of total synergistic (green), additive (blue), indifferent (hashed grey), and antagonistic (orange) reported interactions for (A) Gram-negative and (B) Gram-positive bacteria. White boxes indicate interactions not appropriately assessed for synergism.

(A)	Gram-Negative	*Total*		(B)	Gram-Positive	*Total*
	*Acinetobacter baumannii*	20			*Bacillus cereus*	1
		50				1
		14			*Bacillus subtilis*	1
		7				3
	*Aeromonas hydrophila*	2			*Cutibacterium acnes*	3
	*Aeromonas sobria*	1			*Clostridium dificile*	2
	*Burkholderia pseudomallei*	1			*Enterococcus faecium*	10
	*Cronobacter sakazakii*	1				11
	*Enterobacter cloacae*	2				2
		2				3
	*Escherichia coli*	43				13
		57			*Enterococcus faecalis*	5
		12				10
		1				3
		11				2
	*Klebsiella pneumoniae*	31				6
		24			*Mycobacterium bovis*	1
		12			*Micrococcus luteus*	7
		11				28
	*Moraxella catarrhalis*	1				1
	*Pseudomonas aeruginosa*	80			*Mycobacterium smegmatis*	1
		83			*Mycobacterium tuberculosis*	1
		31				1
		24			*Staphylococcus aureus*	65
	*Pseudomonas fluorescens*	14				90
	*Pseudomonas mirabilis*	1				25
		1				7
	*Pseudomonas putida*	1				42
	*Pseudomonas vulgaris*	1			*Staphylococcus epidermidis*	7
		1				2
	*Salmonella enterica* serotype Typhimurium	6				1
		11				4
		2			*Staphylococcus faecalis*	1
		1				1
		3			*Streptococcus mutans*	2
	*Vibrio anguillarum*	1				1
	*Vibrio cholerae*	1				3
	*Vibro harveyi*	1			*Streptococcus* spp.	1
		1				1
	*Vibro parahaemolyticus*	1				1
		1				
	*Vibrio splendidus*	1				
	*Vibrio vulnificus*	1				
	*Yersinia enterocolitica*	1				

**Table 3:** Reported antibacterial classes and their combinatory HDP–antibiotic activity total. Reported interactions are denoted as synergistic (green), additive (blue), indifferent (hashed grey), and antagonistic (orange). White boxes indicate interactions not appropriately assessed for synergism. Antibiotic abbreviations: AG, aminoglycosides; AM, amphenicols; CP, carbapenems; CS, cephalosporins; FQ, fluoro/quinolones; GP, glycopeptides; ML, macrolides; PC, penicillins; PG, phosphoglycolipids; PM, polymixins; PP, polypeptides; RF, rifamycins; TC, tetracyclines.

*FICI Assessment*	AG	AM	CP	CS	FQ	GP	ML	PC	PG	PM	PP	RF	TC	Other	**Total**
*Synergy*	36	23	14	25	33	16	32	47	1	25	0	32	8	12	**304**
*Additive*	48	14	26	22	59	28	27	43	0	34	0	40	14	16	**371**
*Indifferent*	18	3	1	7	7	13	2	26	1	6	2	5	6	9	**106**
*Antagonistic*	0	5	0	0	3	1	0	0	0	1	3	0	1	0	**14**
*Not Assessed*	17	10	6	10	7	33	4	41	0	9	0	2	1	7	**147**
**Total**	**119**	**55**	**47**	**64**	**109**	**91**	**65**	**157**	**2**	**75**	**5**	**79**	**30**	**44**	**942**

**Table 4:** HDPs and their derivatives discussed in this review that have progressed to clinical trials. Origin peptide and species, clinical use investigated, clinical trial phase and progression, and DRAMP database designation [12] are detailed for each HDP therapy also found in the investigation of synergistic interactions.

**Kingdom:** **Animalia** *Phylum:* *Chordata* Order	Origin Species	Peptide	Derivative	Clinical Use	Clinical Trial Phase	DRAMP ID
Amphibia	*Xenopus laevis*	Magainin 2	Pexiganan	Diabetic foot ulcer infection	III Failed	18057
Artiodactyla	*Bos taurus*	Bactenecin	IDR-1	Immuno-compromised infection	I	18178
		Bactenecin, Indolicidin	IMX942	Nosocomial infection, chemotherapeutic-induced neutropenia	II	18152
		Indolicidin	Omiganan	Rosacea, atopic dermatitis, acne vulgaris, genital warts	III	18160
	*Sus domesticus*	Protegrin-1		Pneumonia peritoneal infection	-	18073
			Iseganan	Ventilator-associated pneumonia, opportunistic infection during radiotherapy	III Failed	18059
			Murepavadin	Nosocomial and ventilator-associated pneumonia	III Suspended	20774
Primates	*Homo sapiens*	Cathelicidin		Venous leg ulcers	I–II	18084
			AMP60.4Ac	Chronic suppurative otitis media	II	18161
		Defensin	Brilacidin	Acute bacterial skin infection	IIb	18158
		α-MSH	CZEN-002	Gram-positive and -negative bacterial and fungal infection	IIb	18083
			Modimelanotide	Sepsis, acute post-surgical kidney injury	II	18164

## Data Availability

The data presented in this study are available within the article or upon request from the corresponding author.
